# Development of parvalbumin-immunoreactive neurons in the postnatal human hippocampal formation

**DOI:** 10.3389/fnana.2023.1058370

**Published:** 2023-02-02

**Authors:** Hajnalka Ábrahám, Hisae Kojima, Katalin Götzer, Abigél Molnár, Tamás Tornóczky, László Seress

**Affiliations:** ^1^Department of Medical Biology and Central Electron Microscopic Laboratory, University of Pécs Medical School, Pécs, Hungary; ^2^Center for Neuroscience, University of Pécs, Pécs, Hungary; ^3^Institute for the Psychology of Special Needs, Bárczi Gusztáv Faculty of Special Needs Education, Eötvös Loránd University, Budapest, Hungary; ^4^Department of Pathology, University of Pécs Medical School, Pécs, Hungary

**Keywords:** interneuron, basket cell, hippocampus, dentate gyrus, memory

## Abstract

**Introduction:** Parvalbumin (PV) is a calcium-binding protein present in fast-spiking GABAergic neurons, such as basket and axo-axonic cells. Previous studies in non-human primates reported prenatal expression of PV in the temporal archicortex including entorhinal cortex and hippocampal formation. In contrast, PV-immunoreactivity was observed only postnatally in the human entorhinal cortex. Regarding PV expression in the human hippocampal formation, no information is available.

**Methods:** In this study, the neurochemical maturation of PV-immunoreactive interneurons was studied in the postnatal developing human hippocampal formation.

**Results:** Before birth, no PV-immunoreactive neurons could be detected in the human hippocampus. At birth, only a few PV-immunoreactive neurons were visible in Ammon’s horn. The first PV-immunoreactive cells in the hilus of the dentate gyrus appeared at the age of 1 month. Even at the age of 5 months, only a few PV-immunopositive cells were present in the dentate hilus. The number of cells and their dendritic and axonal arborization in Ammon’s horn and in the dentate gyrus gradually increased with age. Even at the age of 2 years, dendritic tree and axons of PV-immunoreactive neurons were less complex than can be seen in 8 and 11 years old children.

**Discussion:** Our results showed that long-lasting maturation of PV-immunoreactive interneurons follows the developmental sequence of the subfields of the human hippocampal formation and provides further morphological evidence for the long-lasting functional maturation of the human cortex.

## Introduction

Inhibitory interneurons play an important role in synchronized oscillations in cortical microcircuits (Wang and Buzsáki, 1996; Wallenstein and Hasselmo, 1997). Among the various GABAergic cell types calcium-binding protein Parvalbumin (PV)-expressing cells, the major GABAergic component of the hippocampal intrinsic inhibitory circuitry, are powerful regulators of neuronal activity. Fast-spiking PV-immunoreactive neurons are involved in basic microcircuit functions such as feed-forward or feed-back inhibition, and in gamma frequency (30–80 Hz) oscillations (Whittington et al., 1995; Ylinen et al., 1995; Tamas et al., 2000; Freund, 2003; Fuchs et al., 2007; Sohal et al., 2009; Antonoudiou et al., 2020). In addition, PV-immunoreactive neurons are cellular components of complex network operations, including modulation of place and grid field, pattern separations, phase precession, and gain modulation of sensory responses (Hu et al., 2014).

Alteration of PV-immunoreactive cell number and/or neuronal activity in various areas of the brain has been shown in animal models of various diseases such as schizophrenia, temporal lobe epilepsy, stress, autism, and Alzheimer’s disease (Boksa et al., 2016; Drexel et al., 2017; Hashemi et al., 2017; Cameron et al., 2019; Hijazi et al., 2020; Paterno et al., 2021). Abnormal PV expression has also been reported in animal models of autism spectrum disorder (Filice et al., 2016; Lauber et al., 2018). In addition to the animal models, changes in PV expression in the brain of patients with neuropsychiatric or neurodegenerative disorders have been reported (Knable et al., 2004; Rajkowska et al., 2007). In individuals with autism, an increase in PV-immunoreactive interneurons in hippocampal areas CA1 and CA3 has been detected when compared with controls (Lawrence et al., 2010). In temporal lobe epilepsy, loss of PV-immunoreactive cells has been reported in highly vulnerable areas of the Ammon’s horn and the dentate gyrus, though, no correlation has been found between total neuronal loss and PV-immunoreactive neuronal loss in any of the hippocampal fields (Sloviter et al., 1991; Andrioli et al., 2007). In addition, morphological and neurochemical reorganization of PV-immunoreactive axon terminals were reported in the human sclerotic hippocampus, despite the unaltered distribution and numbers of perisomatic inhibitory axon terminals around pyramidal cells of CA1 and granule cells of the dentate gyrus (Wittner et al., 2001, 2005; Arellano et al., 2004; Ábrahám et al., 2020).

Development of PV-immunoreactive basket and axo-axonic cells in the hippocampal formation reveals significant differences in rodents and in non-human primates. While in rodents most of the hippocampal excitatory and inhibitory neurons reveal rapid pre- and postnatal neurochemical as well as morphological development and reach adult-like morphology in the first postnatal week, PV-containing interneurons display long-lasting neurochemical development. These neurons start to express PV only at the end of the first postnatal week in the rodent Ammon’s horn and PV mRNA cannot be detected in the dentate gyrus before postnatal day 12 (Nitsch et al., 1990; Jiang et al., 2001). PV-immunoreactivity reaches an adult-like pattern not before the third week of age, which indicates long-lasting maturation of PV-positive basket and axo-axonic (chandelier) cells in the rodent hippocampal formation.

In contrast, Berger et al. (1999) reported early expression of PV in the hippocampal formation of the macaque monkey, with the appearance of the first PV-containing cell on embryonic day 83 followed by an area-specific developmental sequence. As a result, PV-immunoreactive basket and chandelier cells reach their neuronal targets several weeks before birth in non-human primates. PV has been reported to be prenatally detectable simultaneously in interconnected subfields of the hippocampal formation and the entorhinal cortex, and prenatal maturation of PV-immunoreactive cells has been found in other limbic areas such as the retrosplenial cortex. However, compared to other neurochemical markers of hippocampal cells, PV expression is delayed until the last quarter of gestation (Berger and Alvarez, 1996; Berger et al., 1997, 1999). Due to the functional significance of PV-immunoreactive fast-spiking neurons, the behavioral consequence of prenatal PV-positive neuronal maturation as early hippocampal-dependent memory formation was suggested in both primates and in humans (Diamond, 1990; Berger et al., 1999).

In contrast to the early neuronal maturation found in the monkey, in the human hippocampal formation a long-lasting postnatal development of dendrites and axons of granule cells as well as mossy cells has been reported (Seress and Mrzljak, 1992; Seress et al., 2001; Ábrahám et al., 2009). In the human entorhinal cortex, PV- immunoreactive neurons and processes were virtually absent at birth, and their presence increased gradually from the 5th postnatal month and afterwards (Grateron et al., 2003). This indicates that neurochemical maturation of PV-containing basket cells and axo-axonic neurons is a postnatal event in the human hippocampal formation, and we propose a prolonged dendritic and axonal maturation of these cells. Therefore, in the present study, we examined the postnatal development of PV-immunoreactive neurons in human hippocampal formation.

## Materials and methods

### Tissues of patients used in the study

Hippocampal formations from developing human brains were used in this study. We included the brains of only those individuals whose death was not related to genetic disorders, head injury, or neurological diseases. In any of the cases, the autopsy did not reveal periventricular leukomalacia and the clinical history did not register seizures. The age at death, and cause of death are summarized in Table 1. Autopsies and the removal of the tissue blocks from the brain as well as fixation were performed 6–12 h after death in the Department of Pathology of University of Pécs Medical School. One-2 cm wide blocks were cut from the hippocampal formation and fixed in 4% paraformaldehyde buffered with phosphate buffer (0.1 M PB, pH 7.4) for 10–14 days as described previously (Seress et al., 2001; Ábrahám et al., 2004). During the whole procedure, the regulations of the Hungarian Ministry of Health as well as the policy of the Declaration of Helsinki were followed. Regional and Local Research Ethics Committee of the University of Pécs did not require the study to be reviewed or approved because this study uses deidentified human samples which were obtained as anonymized by-products from a routine pathological autopsy performed in the Department of Pathology of University of Pécs Medical School.

**Table 1:** Personal data and diagnosis verified by autopsy of individuals included in this study.

Cases	Postconceptual age at death	Cause of death	Number of paraffin sections analyzed
1.	28 weeks	CRI, IRDS	3
2.	29 weeks	CRI, IRDS	2
3.	33 weeks	CRI	2
4.	35 weeks	CHD, IRDS	2
5.	37 weeks	BPD	3
6.	39 weeks	CRI, RDS	3
7.	39 weeks	pneumonia, A	2
8.	40 weeks	CRI	3
9.	40 weeks	RDS, A	3
10.	40 weeks	CHD	1
11.	41 weeks	PVT	2
12.	41 weeks	CHD	3
13.	42 weeks	CRI	3
14.	1 postnatal month	CRI	4
15.	1 postnatal month	CRI	3
16.	3 postnatal months	CRI, sepsis	1
17.	3 postnatal months	CHD	2
18.	3 postnatal months	CHD	1
19.	5 postnatal months	SIDS	3
20.	5 postnatal months	RDS, A	3
21.	8 postnatal months	CRI	3
22.	8.5 postnatal months	Pneumonia	2
23.	2 years	Wilms tumor	4
24.	2 years	Pneumonia, sepsis	2
25.	3 years	Penumonia	3
26.	8 years	ALL	3
27.	11 years	ALL	4
28.	47 years	Heart attack	-
29.	53 years	Heart attack	-

Abbreviations: A, asphyxia; ALL, acute lymphoid leukaemia; BPD, broncho-pulmonary dysplasia; CHD, congenital heart disease; CRI, cardio-respiratory insufficiency; IRDS, infant respiratory distress syndrome; PVT, Pulmonal vein transposition; RDS, respiratory distress syndrome; SIDS, sudden infant death syndrome.

### Parvalbumin immunohistochemistry on paraffin sections

Because of the high water content and fragility of peri- and postnatal human brain including the temporal lobe due to the immaturity of the region (e.g., immature myelination), tissue blocks of all children listed in Table 1 were embedded in paraffin and 10 μm thick sections were cut and mounted on gelatin-coated slides. Following deparaffinization and rehydration of the sections, microwave antigen retrieval was performed as described previously (Seress et al., 2001; Ábrahám et al., 2001). Briefly, following washes with Tris buffer (TB, pH 7.6) three times for 10 min, slides were placed in 80 ml plastic jars filled with citrate buffer (pH 6.0) and heated in a microwave oven operating at a frequency of 2.45 GHz and 800 W power setting. After three heating cycles of 5 min each, slides cooled down at room temperature and were repeatedly washed in TB. For visualization of immunoreactive profiles under the light microscope, after washing, sections were pre-incubated in blocking normal horse serum (10% in TB, Vector Laboratories, Burlingame, CA) for 1 h. This step was followed by overnight incubation in the primary polyclonal rabbit anti-PV antibody (1:5,000, Swant, Bellinzona, Switzerland) applicable on paraffin sections in a humidified chamber at room temperature. After washing three times for 10 min binding sites were visualized with biotinylated secondary antibody (1:100, 2 h at room temperature) and with the avidin-biotin-peroxidase detection system (1:50, Universal Vectastain ABC Elite Kit, Vector Laboratories, Burlingame, CA). Using chromogen 3,3’-diaminobenzidine (DAB, 0.04%) and peroxidase substrate hydrogen-peroxide (0.003%) diluted in TB, immunoreaction was carried out under visual control with a light microscope and stopped by the removal of the DAB followed by washes in TB. Following the immunocytochemistry, sections were counterstained with cresyl-violet, dehydrated, cleared with xylene, and covered with DePeX (Fluka, Switzerland). Control sections were treated similarly, except that the primary antiserum was omitted from the procedure. The specificity of anti-PV antiserum has already been determined by the company. Furthermore, if the primary antibody was omitted no immunostaining was observed. Paraffin sections followed by PV-immunohistochemistry were used to analyze the density, the size of the somata, and the dendritic tree of PV-immunoreactive neurons. In addition, the distribution of PV-immunoreactive cells in the hippocampal formation was illustrated by Camera ludica drawings.

### Parvalbumin immunohistochemistry on free-floating vibratome sections

For better visualization and study of the development of PV-ir neurons’ axonal arborization 80 μm thick free-floating sections were cut using a vibratome from postnatal and older infants’ and children brains, as well as from adults (47 and 53 years old). Following fixation, tissue blocks of 1- and 3-month-old and in 2-, 8-, and 11-year-old children were sectioned and individual serial free-floating sections were collected and processed for immunohistochemistry.

Sections were washed in TB and pretreated with a solution of 1% hydrogen peroxide for 30 min to block endogenous peroxidase, followed by pre-incubation in 1% normal horse serum in TB containing 0.4% Triton X-100 (Sigma-Aldrich, Hungary) for 1 h. This step was followed by incubation with primary monoclonal mouse anti-PV antibody (1:5,000, Swant, Bellinzona, Switzerland) applicable on free-floating sections for 72 h at 4°C. Binding sites of the primary antibodies were visualized with biotinylated secondary anti-mouse antibody and the avidin–biotin peroxidase detection system (Universal Vectastain ABC Elite Kit, Vector, Burlingame, CA). The chromogen used was 3,3’-diaminobenzidine (DAB). The tissue sections were then mounted on glass slides, air-dried, ethanol series-dehydrated, cleared with xylene, and cover-slipped with DePeX (Fluka, Switzerland). Parts of PV-immunostained sections were counterstained with cresyl-violet before dehydration. Immunohistochemical control sections were handled in a similar manner, except that primary antibodies were omitted. The specificity of anti-PV antiserum has already been determined by the company. Furthermore, if the primary antibody was omitted no immunostaining was observed.

### Quantitative analysis of PV-immunoreactive cells and dendrites on paraffin sections

**Figure 1:**
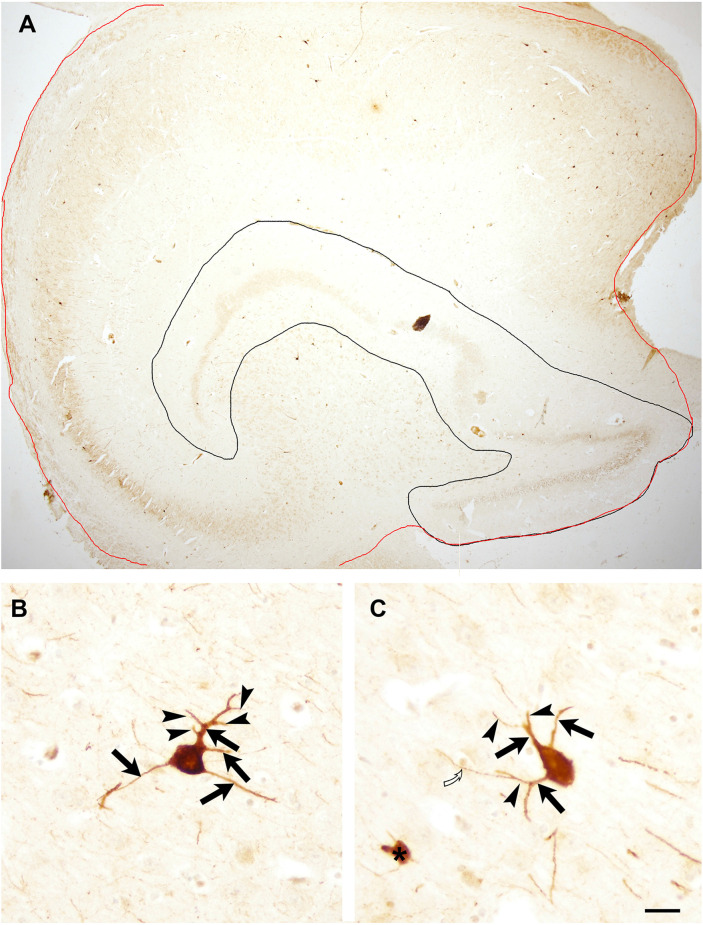
**(A)** Low magnification photomicrograph of the entire hippocampal formation of an 11 years old child to illustrate areas used for quantification. The red line outlines the hippocampal formation, black line encircles the dentate gyrus. Both areas were measured with iTEM software. The area of Ammon’s horn was determined following the subtraction of the dentate gyrus’ area from the area of the entire hippocampal formation. Panels **(B)** and **(C)** illustrate PV-immunoreactive neurons in an 11-year-old child’s section with several primary and secondary dendrites. Arrows point to primary dendrites, arrowhead indicates secondary dendrites. Open curved arrow points to a tertiary dendrite that was not included in the quantification. Asterisk indicates a PV-immunoreactive profile that was excluded from the quantification of immunopositive neurons. Scale bar = 500 μm in **(A)** and 25 μm in **(B)** and **(C)**.

Determination of the number of PV-immunoreactive cells/area was performed on low magnification digital photos taken with an Olympus BX50 light microscope using objectives of 1.2× and 2× magnifications. On these low magnification photos, borders of the whole hippocampal formation and the dentate gyrus were outlined (similarly as shown in Figure 1A) and areas of these structures were determined using iTEM software (Olympus). The area of Ammon’s horn was determined by subtracting the dentate gyrus’ area from the area of the entire hippocampal formation. PV-immunoreactive cells were examined in Ammon’s horn and in the dentate gyrus separately under a light microscope using objectives of 10× and 20× magnification, and PV-immunostained neurons were counted, then cell number/area (number/mm^2^) was determined in each section.

Using high (20× and 40×) magnification, the size of the somata of the cells and their dendritic tree were analyzed. The size of cell bodies was determined by measuring the longest diameter seen in PV-immunoreactive histological preparations using a calibrated ocular micrometer. Soma of each PV-immunopositive neuron found in the hippocampal formation was measured.

Regarding dendritic trees, the number of primary and secondary dendrites were counted in each PV-positive neuron. Figures 1B,C illustrate the criteria for primary and secondary dendrites used for quantification. The percentage of PV-positive cells having 0–5 main dendrites as well as neurons with 0–5 secondary dendrites in the entire hippocampal formation, in Ammon’s horn and in the dentate gyrus was determined. Following quantification, average ± SD of each age group was calculated.

### Statistical analysis

Pearson’s correlation analysis was performed to investigate the association between age and the data obtained by quantification including (i) total neuronal number; (ii) density; (iii) the proportion of primary dendrites; and (iv) soma size of PV-immunoreactive neurons. Statistical significance was set at *p* ≤ 0.05.

## Results

### Morphological observations

Expression of the calcium-binding protein PV in the human postnatal hippocampal formation starts relatively late during development. Before the age of 39–40 gestational weeks, in prematurely born children no PV immunoreactivity could be detected in the human hippocampal formation indicating that basket and axo-axonic cells did not express PV in the late fetal period.

**Figure 2:**
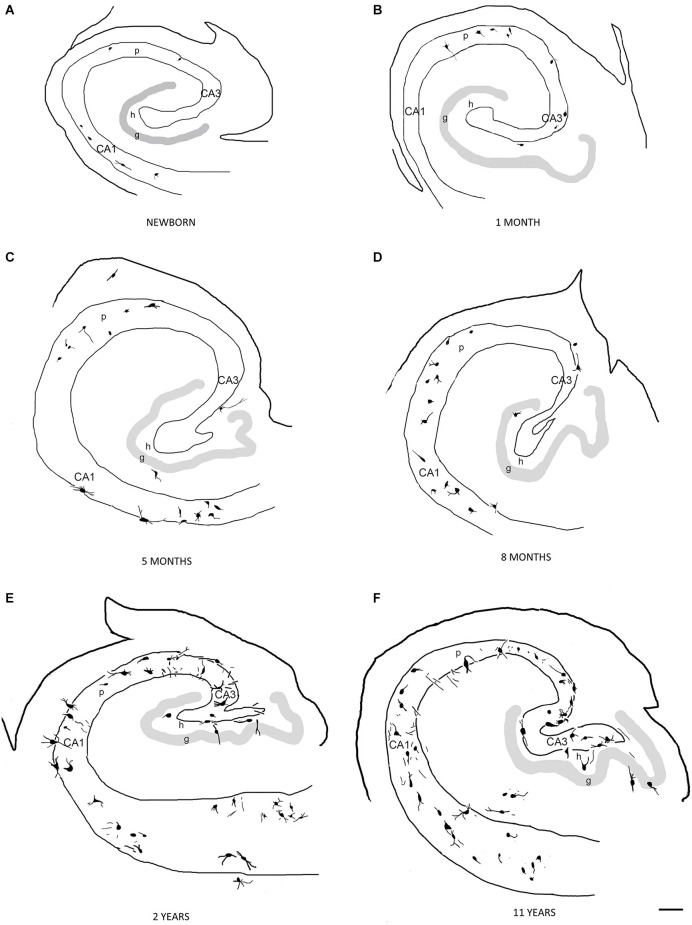
Schematic drawing demonstrating the distribution of PV-immunoreactive neurons in the human hippocampal formation in a newborn **(A)**, in 1 month **(B)**, in 5 **(C)**, and 8 **(D)** months, in 2 **(E)**, and 11 **(F)** years old child. Scale bar = 500 μm in **(A–C)**, 280 μm in **(D)** and **(E)**, and 250 μm in **(F)**. Abbreviations: g, granule cell layer; h, hilus of the dentate gyrus; p, pyramidal layer of CA1, CA3, regions of Ammon’s horn.

**Figure 3:**
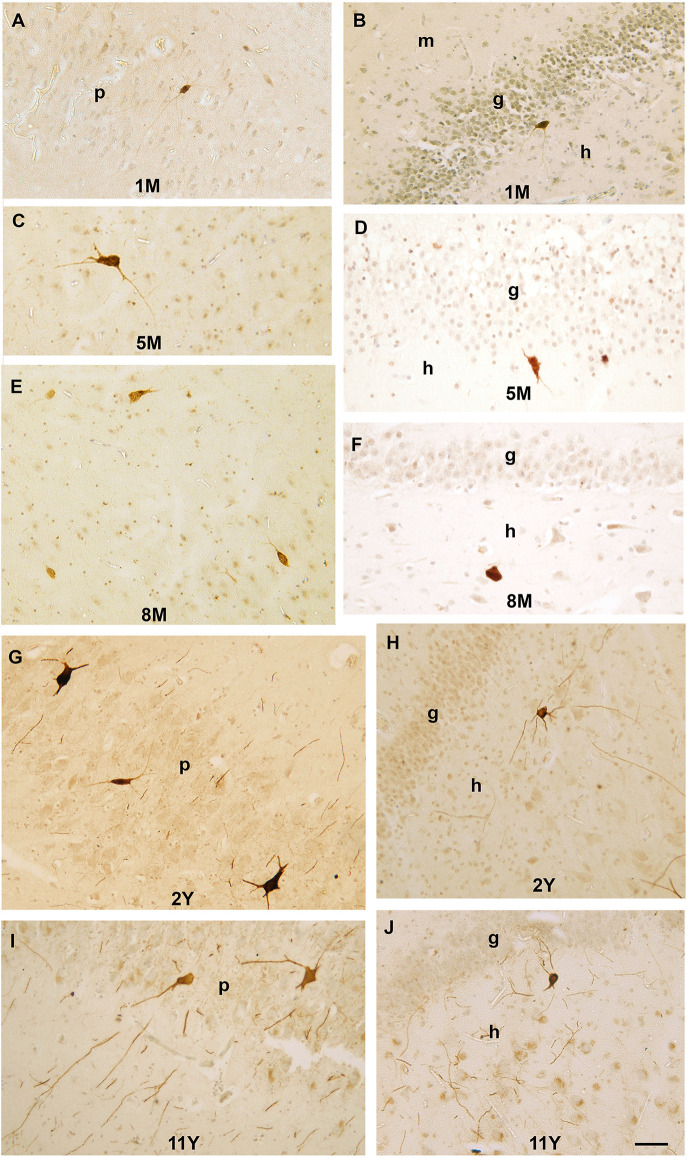
Photomicrographs showing PV-immunoreactive neurons during postnatal development of the human hippocampal formation. **(A)** A PV-immunoreactive cell with immature morphology and thin dendrites in the pyramidal layer of CA3 region in a 1-month-old infant. **(B)** A small PV-immunoreactive cell with immature morphology in the subgranular layer of the dentate gyrus. **(C)** Large PV-immunoreactive neuron with several dendrites next to the CA1 pyramidal layer in a 5-month-old infant. **(D)** A PV-immunoreactive cell in the subgranular layer of the dentate gyrus at the age of 5 months. **(E)** PV-immunoreactive neurons next to the CA3 pyramidal layer in an 8-month-old infant. **(F)** A PV-immunoreactive cell in the dentate hilus in an 8-month-old infant. **(G)** Large PV-immunoreactive neurons and several sections of PV-positive dendrites in the CA1 pyramidal layer of a 2-year-old child. **(H)** Large PV-immunoreactive neurons with several long dendrites in the hilus of the dentate gyrus in a 2-year-old child. **(I)** Large PV-immunoreactive neurons and several sections of PV-positive dendrites in the CA1 pyramidal layer of an 8-year-old child. **(J)** PV-immunoreactive neuron and sections of PV-positive dendrites in subgranular position of an 8-year-old child. Scale bar = 50 μm. Abbreviations: g, granule cell layer; m, molecular layer; h, hilus of the dentate gyrus; p, pyramidal layer of CA1, CA3, regions of Ammon’s horn.

The first PV-immunoreactive cells could be detected at birth (39–40 gestational weeks) in Ammon’s horn and they could be found in the pyramidal layer and the str. oriens of Ammon’s horn (Figure 2A). In the dentate gyrus, PV-immunopositive cells were not yet visible at birth (Figure 2A). In a 1-month-old infant, the first PV-immunoreactive cells could be found in the dentate gyrus along with increasing numbers of PV-positive neurons in the Ammon’s horn of the hippocampal formation (Figures 2B, 3A,B). In the next few months, the number of PV-immunoreactive neurons increased, particularly in the Ammon’s horn (Figures 2C,D, 3C–F), and the cells had more and longer dendrites. Further development could be observed at 2 years and onwards and the number of PV-immunoreactive neurons was increased in both the CA1 and CA3 regions as well as in the dentate gyrus (Figures 2E, 3G,H). Morphology of PV-immunoreactive neurons showed further maturation with their numerous, relatively long and arborized dendrites that were the most complex at the age of 11 years (Figures 2F, 3I,J).

**Figure 4:**
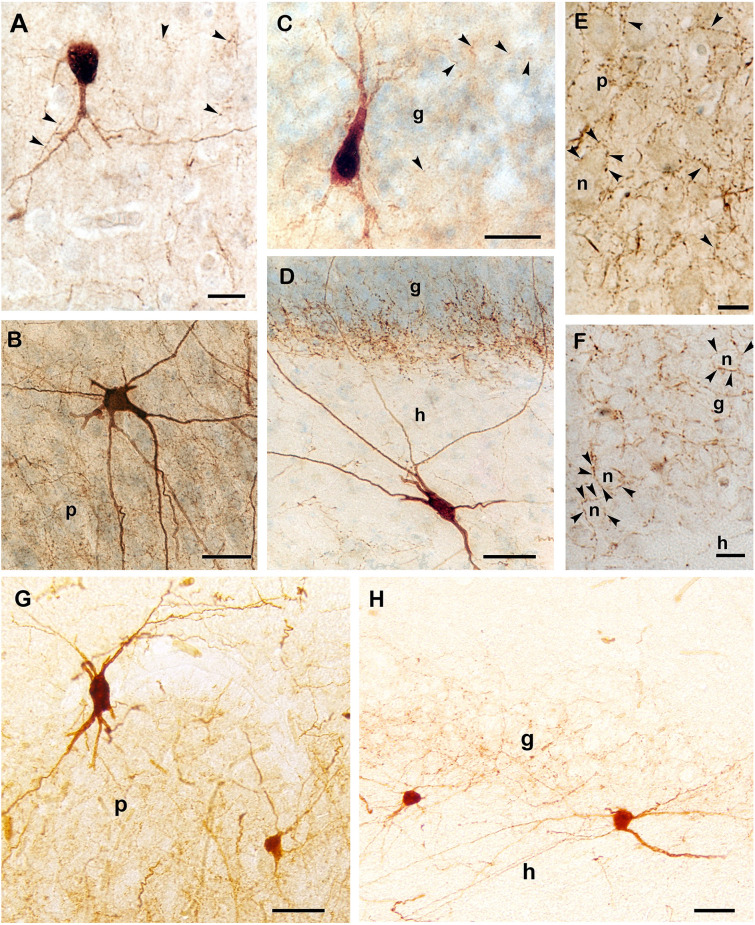
PV-immunoreactive interneurons and axons in the hippocampal formation of a 1-month-old **(A)**, a 3-month-old **(C)**, and in an 11-year-old **(B,D–F)** child. **(A)** PV-positive cell with immature morphology and sparse dendritic branches in a 1-month-old infant. **(B)** Large, multipolar PV-positive cell in the pyramidal layer of Ammon’s horn of an 11-year-old child. The PV-positive axonal network is confined to the pyramidal cell layer. **(C)** Soma and main dendrites of a PV-immunoreactive cell in the granule cell layer of the dentate gyrus in a 3-month-old infant. **(D)** A large PV-immunoreactive hilar (h) neuron with long dendrites that cross the granule cell layer of an 11-year-old child. Inside the granule cell layer, the dense PV-immunoreactive axonal network appears to be denser at the hilar border. The density of axonal branches is low in the hilus that corresponds with a lower cellular density in the hilus than in the granule cell layer. **(E)** High magnification photomicrograph of the PV-positive axonal network in the pyramidal layer. **(F)** Large magnification photomicrograph of PV-immunoreactive axon terminals in the granule cell layer of an 11-year-old child. Arrowheads point to PV-immunoreactive axonal swellings (terminal-like boutons). The axons (arrowheads) display large numbers of boutons that appear to surround somata of pyramidal **(E)** and granule cells **(F)**. **(G)** PV-immunoreactive cells and axonal plexus in the CA1 pyramidal layer of an adult. **(H)** PV-immunoreactive neurons in the subgranular layer of the dentate gyrus in an adult. Boutons of PV-immunoreactive axons are visible in the granule cell layer of the dentate gyrus. Scale bars = 10 μm in **(F)**, 20 μm in **(A)** and **(E)**, 25 μm in **(C)**, and 50 μm in **(B,D,G)**, and **(H)**. Abbreviations: g, granule cell layer; h, hilus of the dentate gyrus; n, neuronal soma; p, pyramidal layer of Ammon’s horn.

Axonal development of PV-immunoreactive neurons was studied on thicker (80 μm) sections in 1- and 3-month-old infants and in 2-, 8- and 11-year-old children. Despite the poor dendritic tree present in the 1 and 3-month-old infants, a few PV-positive axons and a few axon-terminal-like boutons could already be observed in these months (Figures 4A,B). According to the development described above, dendritic maturation of PV-immunoreactive neurons occurs along the continuously increasing axonal arborization in the next few months. Even in a 2-year-old child, axonal branching and the number of terminal boutons along with the above-mentioned dendritic tree are less developed than in 8 or 11 years old children. The morphology of PV-immunoreactive cells and the pattern of their axonal network in the principal cell layers of Ammon’s horn and the dentate gyrus at the age of 11 years was comparable with that could be seen in the adult hippocampal formation (Figures 4B–H).

### Quantitative analysis of PV-immunoreactive cells and dendrites

**Figure 5:**
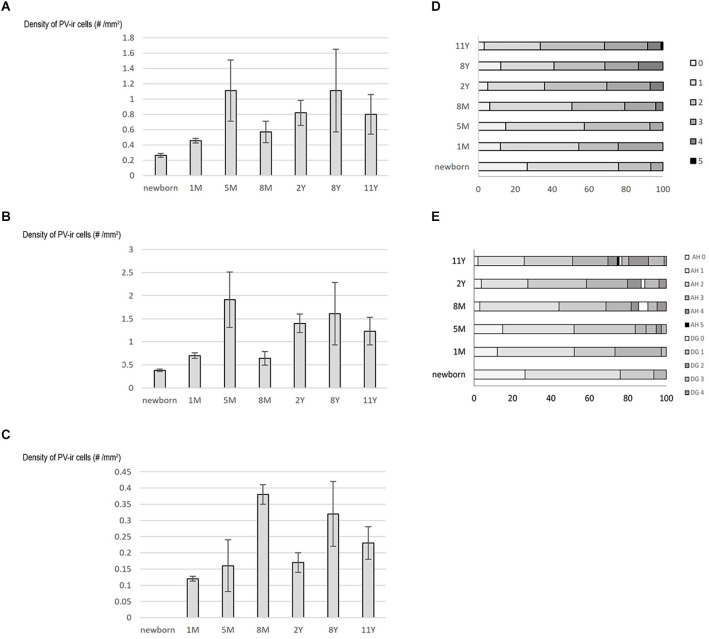
Graphs demonstrating results of quantification on PV-immunoreactive neurons in the developing hippocampal formation. Density (cell number/1 mm^2^ area) of PV-immunoreactive neurons is shown in the whole hippocampal formation **(A)**, in Ammon’s horn **(B)**, and in the dentate gyrus **(C)**. **(D)** and **(E)** Graphs demonstrate data on dendritic development of the PV-immunoreactive neurons in the entire hippocampal formation **(D)** and in the Ammon’s horn and dentate gyrus separately **(E)**. Percentage of PV-immunoreactive neurons with 0–5 primary dendrites shown in different age groups. Abbreviations: AH, Ammon’s horn; DG, dentate gyrus; M, month; PV-ir, PV-immunoreactive; Y, years.

Following full-term birth, the total number, as well as the density (cell number/area) of PV-positive neurons, was low in Ammon’s horn, while cells did not appear in the dentate gyrus (Table 2, Figures 5A–C). In the first postnatal month, the total number and density of cells slightly increased in the entire hippocampal formation including Ammon’s horn, and the first PV-immunoreactive neurons could be observed in the dentate gyrus (Table 2, Figures 5A–C). We could detect the largest cell number/area at the age of 5 months, in the entire hippocampal formation, and in the Ammon’s horn (Figures 5A,B). In addition, the total number of the PV-immunopositive neurons in the Ammon’s horn as well as in the entire hippocampal formation was higher at 5 months than at 8 months of age (Table 2). In the dentate gyrus, however, the total number of PV-immunoreactive cells was higher at 8 months than at 5 months of age (Table 2), and the highest PV-immunoreactive cell density (number/area) was observed at the age of 8 months (Figure 5C). At later developmental stages, though variably, the total number of cells further increased in Ammon’s horn and in the dentate gyrus (Table 2), although, due to the relatively rapid growth of the hippocampal formation after the first year, density values did not follow the changes of the total cell numbers (Figures 5A–C). Pearson’s correlation analysis did not show a significant correlation between age groups and the total number of PV-immunoreactive cells in the entire hippocampal formation and in Ammon’s horn, but in the dentate gyrus, a significant correlation was observed (*p* = 0.02). No significant correlation could be detected between ages and density of PV-immunoreactive neurons either in the entire hippocampal formation or in the two other examined regions, although, a tendency could be seen in the dentate gyrus (*p* = 0.052).

**Table 2:** Summary of the hippocampal areas and cell counts.

Age	Area of the HF (mm^2^)	Area of Ammon’s horn (mm^2^)	Area of dentate gyrus (mm^2^)	Cell # in HF	Cell # in Ammon’s horn	Cell # in dentate gyrus
newborn	21.8 ± 5.96	15.1 ± 6.21	6.7 ± 2.87	4.79 ± 1.3	4.79 ± 1.3	0
1 M	15.0 ± 5.98	8.6 ± 3.67	6.4 ± 2.72	6.3 ± 0.96	5.5 ± 1.02	0.8 ± 0.96
5 M	24.2 ± 6.18	13.4 ± 2.33	11.8 ± 4.85	26.4 ± 12.51	24.6 ± 8.35	1.8 ± 1.52
8 M	21.5 ± 1.94	15.6 ± 3.68	5.9 ± 2.75	12.7 ± 3.21	10.4 ± 4.18	2.3 ± 0.57
2 Y	43.4 ± 11.19	23.1 ± 6.18	20.3 ± 5.08	35.1 ± 3.59	31.7 ± 3.09	3.4 ± 0.58
8 Y	38.9 ± 6.28	22.7 ± 1.5	16.2 ± 3.13	42.0 ± 14.44	36.8 ± 13.81	5.2 ± 1.52
11 Y	42.1 ± 8.64	23.8 ± 5.18	18.3 ± 4.22	33.7 ± 5.51	29.4 ± 6.86	4.3 ± 1.51

In the early postnatal ages, somata of PV-immunopositive cells were small and they had poorly developed dendritic tree, which indicates the immaturity of these neurons (Figures 3A,B, 4A,C). Measuring the longest diameter of the cells, fast continuous increase could be seen in the size (Table 3). Interestingly, the smallest size was detected at 8 months of age. Despite this, Pearson’s correlation analysis revealed significant (*p* = 0.046) correlation between ages and the size of the soma of hippocampal PV-immunoreactive neurons.

**Table 3:** Average ± SD of the diameter of PV-immunoreactive neurons in the Ammon’s horn in different age-groups.

Newborn	42GW	1M	5M	8M	2Y	8Y	11Y
21.75 ± 2.66	21.82 ± 3.4	22.98 ± 3.11	22.73 ± 7.68	22.41 ± 2.46	26.77 ± 1.48	26.48 ± 4.59	28.63 ± 2.78

The dendritic tree of PV-immunoreactive cells was analyzed by determination of the proportion of cells with primary and secondary dendrites in different age groups. Parallel with the age, the proportion of PV-immunoreactive neurons with primary dendrites increased in the entire hippocampal formation as well as in both Ammon’s horn and the dentate gyrus (Figures 5D,E). A tendency could be observed regarding the proportion of cells with 0 and 1 dendrites that proportion decreased with the age, while the complexity of the dendritic tree increased with the increasing number of cells with four and five main dendrites. Pearson’s correlation analysis showed a significant positive correlation (*p* = 0.013) between ages and the proportion of PV-immunoreactive cells with two dendrites in the dentate gyrus.

The morphology of PV-immunoreactive cells showed further maturation with their numerous, relatively long, and arborized dendrites. Analysis of numbers of the secondary dendrites revealed a tendency indicating the association between the age and the proportion of PV-immunoreactive neurons with higher numbers of main and secondary dendrites (Table 4). Despite the advanced maturation, the morphology of the dendrites and their branching indicated that PV-immunoreactive cells were less developed in the 2-year-old child than in 8- and 11-year-old children (Table 4). Quantification confirmed the further ramification of dendrites of PV-immunoreactive neurons between the ages of 8 and 11 years (Table 4).

**Table 4:** Proportion of PV-immunoreactive cells with different numbers of main and secondary dendrites.

	Newborn	1M	5M	8M	2Y	8Y	11Y
cells with 1 main dendrite without secondary dendrite	0.26	0.12	0.087	0	0.14	0.037	0.118
cells with 1 main dendrite with 1 secondary dendrite	0	0.04	0.037	0	0.054	0.05	0.052
cells with 1 main dendrite with 2 secondary dendrites	0	0.046	0.043	0	0.022	0.25	0.005
cells with 1 main dendrite with 3 secondary dendrites	0	0	0	0.11	0.032	0.13	0.008
cells with 2 main dendrites without secondary dendrite	0.125	0.103	0.2	0.33	0.104	0.096	0.237
cells with 2 main dendrites with 1 secondary dendrite	0.042	0.093	0.13	0.11	0.122	0.195	0.135
cells with 2 main dendrites with 2 secondary dendrites	0.062	0.014	0	0	0.131	0.013	0.04
cells with 2 main dendrites with 3 secondary dendrites	0.03	0.014	0.028	0	0.051	0	0.01
cell with 2 main dendrites with 4 secondary dendrites	0	0	0	0	0.01	0	0.008
cells with 3 main dendrites without secondary dendrite	0.103	0.187	0.22	0.11	0.116	0.038	0.087
cells with 3 main dendrites with 1 secondary dendrite	0.073	0.055	0.09	0	0.069	0.072	0.105
cells with 3 main dendrites with 2 secondary dendrites	0.095	0.07	0.018	0	0.05	0.063	0.059
cell with 3 main dendrites with 3 secondary dendrites	0	0.029	0	0	0.029	0.038	0.013
cells with 3 main dendrites with 4 secondary dendrites	0	0	0	0	0.016	0	0.044
cells with 3 main dendrites with 5 secondary dendrites	0.04	0.014	0	0.12	0	0	0.008
cells with 4 main dendrites without secondary dendrite	0.08	0.028	0.046	0.11	0.016	0	0.011
cells with 4 main dendrites with 1 secondary dendrite	0	0.05	0.01	0	0.014	0	0.01
cells with 4 main dendrites with 2 secondary dendrites	0	0.073	0.055	0	0.024	0	0.01
cells with 4 main dendrites with 3 secondary dendrites	0.03	0.05	0.038	0	0	0.013	0.011
cells with 4 main dendrites with 4 secondary dendrites	0.06	0	0	0.11	0	0.013	0.005
cells with 4 main dendrites with 5 secondary dendrites	0	0.014	0	0	0	0	0.005
cell with 5 main dendrites without secondary dendrite	0	0	0	0	0	0	0
cells with 5 main dendrites with 1 secondary dendrite	0	0	0	0	0	0	0.013
cells with 5 main dendrites with 2 secondary dendrites	0	0	0	0	0	0	0
cells with 5 main dendrites with 3 secondary dendrites	0	0	0	0	0	0	0
cells with 5 main dendrites, with 4 secondary dendrites	0	0	0	0	0	0	0.005
	code:	0.01>	0.011–0.05	0.051–0.1	0.11–0.2	0.21–0.3	0.3<

Abbreviations: M, months; Y, years.

## Discussion

In the present work, we have found that expression of PV-immunoreactivity in the human hippocampal formation occurs only after birth and therefore, we suppose that maturation of PV-positive basket and axo-axonic neurons is a long-lasting postnatal process. We have observed that before birth, no PV-immunoreactive neurons could be detected in Ammon’s horn and in the dentate gyrus. At full-term birth, a few PV-positive cells appeared in Ammon’s horn, while PV-containing neurons could be found in the dentate gyrus only after the first postnatal month. These findings are in sharp contrast with previous works that observed prenatal expression of PV-immunoreactive neurons in the non-human primate hippocampus (Berger and Alvarez, 1996; Berger et al., 1999). In macaque monkeys, PV was expressed already at mid-gestation (on embryonic day 83) in Ammon’s horn (Berger and Alvarez, 1996). Berger et al. (1999) also showed that a large population of PV-immunoreactive hippocampal basket cells appears morphologically mature several weeks before birth (Berger et al., 1999). PV-immunoreactive neurons mature fast also in other primate archicortical areas including the entorhinal cortex and retrosplenial cortex (Berger et al., 1997). In contrast, in the human entorhinal cortex, no PV-immunoreactive cells were detected at birth and the first immunopositive neurons appeared only in the first few postnatal months (Grateron et al., 2003). This finding is in harmony with our results about the late expression of PV in human hippocampal formation.

When PV was first expressed at full-term birth, only a few, immature small PV-immunoreactive cell bodies were seen with short, rarely-branching dendrites in Ammon’s horn, and a month later in the dentate gyrus. In a 1-month-old infant, PV-immunoreactive cells still had immature dendritic tree. The morphology of these cells differed from the morphology of PV-immunoreactive cells observed in the adult hippocampal formation (Seress et al., 1993; Ábrahám et al., 2020). In the next few months, both numbers of PV-immunoreactive cells and dendritic and axonal arborization expanded, although the developmental delay in the dentate gyrus, compared to the development of the PV-positive cells in Ammon’s horn is obvious. Even in a 2-year-old child, the morphology of the dendrites and the axonal arborization of PV-immunoreactive neurons was less developed than in 8 or 11 years old children, when these cells reached the morphology observed in adults.

### Technical consideration and limitations of the study

In the present study, we used post-mortem human brain samples of newborns, infants, and children removed by pathological dissection. The major limitation of the study is post-mortem delay, since the longer the time between death and the fixation, the more the alterations could be observed in the tissue. Previous studies indicate that post-mortem delay and temperature influence expression levels of proteins, and therefore, immunohistochemical results (Hilbig et al., 2004; Gonzalez-Riano et al., 2017). Our earlier observations were in accord with this notion, and therefore care was taken to reduce post-mortem delay time. Due to ethical regulations and technical reasons, autopsies and the removal of tissue blocks from the brain as well as fixation were performed 6–12 h after death. Gonzalez-Riano et al. (2017) showed that 5 h long post-mortem delay decreases immunoreactivity and weakens the staining intensity of several proteins, including calbindin and PV. In contrast, Hilbig et al. (2004) reported that calbindin immunoreactivity in the hippocampus was well preserved up to 12 h. We have used tissue samples with 6–12 h long post-mortem delay times in several other studies using different antibodies for the detection of various proteins including calbindin (Seress et al., 2001; Ábrahám et al., 2004, 2009, 2010, 2012). Although, the deleterious effect of post-mortem delay should be considered, these studies indicate the feasibility of human samples’ use with 12 h long or shorter post-mortem delay. In the present study, PV-immunohistochemistry was performed on sections of identical brain samples used in the above-mentioned studies. Only sections with reliable and consistent staining were chosen for the morphological examinations and quantification.

Another limitation of our study is the use of relatively thin paraffin sections that affects the observation and quantification of large three-dimensional structures such as PV-immunoreactive neurons with long arborized dendrites. However, because of the immaturity and the high water-content, peri- and postnatal human brain regions including the hippocampal formation are very fragile. Therefore preservation of the tissue was the most effective using paraffin embedding. We also examined free-floating sections of a few brains, mainly from older children, because in most of the peri- and young postnatal cases vibratome sectioning was not feasible.

A further limitation of our study is the low number of samples that influences statistical analysis. Access to human developing brain tissue is limited, especially that of postnatal, infants, and children samples. In addition, we have to emphasize that only good quality sections were used for quantitative analyses, which further decrease the sample size and the statistical power of our study. Another problem is the heterogeneity of the individuals regarding birth weight, gestational age at birth, morbidity, and the cause of death, along with undisclosed factors such as nutrition and postnatal development as well as genetical and epigenetical changes that all could contribute to the most common accompanying factor of the human studies, which is the large individual variability. Regarding morbidity, different types of medical treatment could potentially influence our results. Unfortunately, all of the children used in this study received various pharmacological treatments according to their medical conditions, since many of them were treated in the intensive care unit before death. The influence of medical drugs and pathological conditions (e.g., epilepsy) on PV immunoreactivity has already been demonstrated (Wittner et al., 2001; Ábrahám et al., 2020; Sitaš et al., 2022), however, one can propose that PV expression was affected similarly in children used in our present study, due to their severe illnesses. The only exception is one of the 5-month old babies who potentially had no previous medication and deceased due to sudden infant death syndrome (SIDS). Interestingly, the density of PV immunoreactive neurons was the highest at 5 months of age that was due to the strong PV expression of the child with SIDS. However, we cannot exclude individual variability behind this phenomenon. An examination of the numbers of neurons in the adult human hippocampal formation revealed large individual variability (Simić et al., 1997). According to this study, a control human person has two or three times larger neuronal number than another control human. Since such difference is normal in adults, we can suggest that the individual variability that we could detect in our study may be common and normal among infants and children.

In summary, the low number of samples combined with the large individual variability of the quantitative data influenced the results of the statistical analyses and might be a reasonable explanation for the lack of significant correlation of variables in certain areas and the positive significant linear correlation of other variables in other areas. The strongest correlation was found between the age of children and the size of PV-immunoreactive cells with the data obtained by measurements of more than 540 cells. In addition, a significant correlation between children’s age and cell number and a tendency of association between age groups and cell density were detected in the dentate gyrus. We have to emphasize that in the dentate gyrus both the number and density of PV-positive cells as well as their variability were smaller than those in Ammon’s horn. These facts signify the importance of sample size and individual variability characteristics of humans.

Despite the limitations described above, we found a clear sequence of the development of PV-immunoreactive neurons, and quantification supported the morphological characteristics observed.

### The prolonged development of human hippocampal PV-neurons and functional implications

The late onset and prolonged morphological maturation of PV-immunoreactive neurons in humans are in contrast to that found in non-human primates, despite the large number of data that would contradict this. Both in monkeys and in humans, neuronal proliferation was reported to cease fetally in Ammon’s horn, and the predominantly prenatal formation of granule cells of the dentate gyrus declines steadily during the first few postnatal months (Rakic and Nowakowski, 1981; Eckenhoff and Rakic, 1988; Seress et al., 2001). By mid-gestation, areal differentiation is already visible both in the human as well as monkey hippocampal formation (Arnold and Trojanowski, 1996). In the fetal monkey, catecholaminergic and serotoninergic projections are present in the hippocampus and the entorhinal cortex (Berger et al., 1993). In humans, reciprocal connections between the entorhinal cortex, Ammon’s horn, and subiculum were found at mid-gestation, although entorhinal projections to the dentate gyrus were sparse (Hevner and Kinney, 1996). In contrast to this, long- lasting postnatal myelination was reported in the human hippocampal formation especially in the dentate gyrus, along with prolonged morphological and neurochemical development of granule cells as well as mossy cells of the dentate gyrus that mature beyond 5 years of age (Seress, 1992; Seress and Mrzljak, 1992; Ábrahám et al., 2009, 2010). Thus, the developmental delay of PV-immunoreactivity in the human dentate gyrus compared to Ammon’s horn observed in this study is in harmony with these above-mentioned observations of long-lasting maturation of the human dentate gyrus.

In our material, the adult-like morphology of PV- immunoreactive cells appeared in 8 and 11 years old children. However, we had no preparation between the 2 and 8 years of age, therefore, we could not exactly predict the time when fully matured PV- immunoreactive neurons are present in the human hippocampal formation. Because of this, we suggest that morphological maturation of these cells is completed during this period (between 2 and 8 years of age), similarly to the development of hilar mossy cells and granule cells of the dentate gyrus (Seress and Mrzljak, 1992; Ábrahám et al., 2009).

Granule cells of the dentate gyrus are important post-synaptic targets of entorhinal afferents that are inevitable in learning and memory. A recent study indicates that the maturation of the entorhinal-hippocampal network follows a stereotypical sequence in postnatal mice and the sequence originated in the medial entorhinal cortex. Along with the maturation of excitatory neurons of the network, maturation-related increase in PV expression across the entire entorhinal-hippocampal complex is influenced by the activity of stellate cells in layer 2 of the medial entorhinal cortex (Donato et al., 2017). The coincidence of the postnatal development of PV-immunoreactive neurons found in the human entorhinal cortex by Grateron et al. (2003) and the prolonged maturation of these cells found by us in the Ammon’s horn and the dentate gyrus, along with other developmental studies indicates the relevancy of the coupled and sequential maturation of the entorhinal-hippocampal network (Liu et al., 2021; Šimić et al., 2022).

In fast spiking interneurons, cytosolic expression of PV has been correlated with their structural and functional maturation (Doischer et al., 2008). While we have to emphasize that PV immunohistochemistry is suitable to detect both axo-axonic cells and fast-spiking basket cells, the role of the latter in hippocampal-dependent memory formation due to their involvement in complex network operations indicates that the maturation of PV-immunoreactive neurons is reflected in learning and memory processes (Whittington et al., 1995; Ylinen et al., 1995; Tamas et al., 2000; Freund, 2003; Fuchs et al., 2007; Antonoudiou et al., 2020; Strüber et al., 2022). In harmony with the early hippocampal development reflected by the predominantly prenatal maturation of PV-immunoreactive neurons in non-human primates, hippocampal-dependent form of recognition memory has been reported soon after birth. This raised the possibility that the entorhinal-hippocampal network may be operational before birth (Bachevalier et al., 1993; Bachevalier and Mishkin, 1994). In humans, Pascalis and de Schonen (1994) reported early face recognition memory after birth, however, other studies revealed that earlier than 2 years of age, children are unable to form and/or store episodic memories for recall later in life (Newcombe et al., 2007). Allocentric spatial competence becomes adult-like between 2–7 years depending on the task used (Newcombe et al., 1998). In addition, children under the age of 4 years could not complete hippocampus-dependent tests in the Morris-water maze or radial-arm maze adopted for humans, and adult-like proficiency could be seen only at the age of 8 years (Overman et al., 1996).

These behavioral findings above are in full harmony with our results showing late postnatal morphological maturation of PV-immunoreactive neurons. In addition to the long-lasting development of granule and mossy cells of the dentate gyrus, as well as the prolonged myelination observed in the human hippocampal formation (Seress and Mrzljak, 1992; Seress et al., 2001; Ábrahám et al., 2009, 2010), our recent results provide further morphological evidence for the long-lasting functional maturation of the human hippocampal formation as well as the human entorhinal-hippocampal network.

## Data availability statement

The raw data supporting the conclusions of this article will be made available by the authors, without undue reservation.

## Ethics statement

Regional and Local Research Ethics Committee of the University of Pécs did not require the study to be reviewed or approved because this study uses deidentified human samples which were obtained as anonymized by-products from routine pathological autopsy performed in the Department of Pathology of University of Pécs Medical School.

## Author contributions

HÁ: writing article, analyses of data, collecting samples, supervision of research. HK: collecting and analysis of data, Camera lucida drawings. KG: collecting and analysis of data, formation of figures. AM: formation of figures, data analyses. TT: collecting samples, contribution in writing article. LS: contribution in writing article, supervision of research. All authors contributed to the article and approved the submitted version.
